# Photochemical solution processing of films of metastable phases for flexible devices: the β-Bi_2_O_3_ polymorph

**DOI:** 10.1038/srep39561

**Published:** 2016-12-20

**Authors:** Dulce Pérez-Mezcua, Iñigo Bretos, Ricardo Jiménez, Jesús Ricote, Rafael J. Jiménez-Rioboó, Cosmelina Gonçalves da Silva, Daniel Chateigner, Luis Fuentes-Cobas, Rafael Sirera, M. Lourdes Calzada

**Affiliations:** 1 Instituto de Ciencia de Materiales de Madrid, Consejo Superior de Investigaciones Científicas (ICMM-CSIC), C/ Sor Juana Inés de la Cruz, 3, Cantoblanco, 28049, Madrid, Spain; 2 Departamento de Química, Facultad de Ciencias, Universidad de Navarra, 31008, Pamplona, Spain; 3 CRISMAT—ENSICAEN and IUT-Caen, Université de Caen Normandie, 14050, Caen, France; 4 Centro de Investigación en Materiales Avanzados, 31109, Chihuahua, Mexico

## Abstract

The potential of UV-light for the photochemical synthesis and stabilization of non-equilibrium crystalline phases in thin films is demonstrated for the β-Bi_2_O_3_ polymorph. The pure β-Bi_2_O_3_ phase is thermodynamically stable at high temperature (450–667 °C), which limits its applications in devices. Here, a tailored UV-absorbing bismuth(III)-*N*-methyldiethanolamine complex is selected as an ideal precursor for this phase, in order to induce under UV-light the formation of a –Bi–O–Bi– continuous network in the deposited layers and the further conversion into the β-Bi_2_O_3_ polymorph at a temperature as low as 250 °C. The stabilization of the β-Bi_2_O_3_ films is confirmed by their conductivity behavior and a thorough characterization of their crystal structure. This is also supported by their remarkable photocatalytic activity. Besides, this processing method has allowed us for the first time the preparation of β-Bi_2_O_3_ films on flexible plastic substrates, which opens new opportunities for using these materials in potential applications not available until now (e.g., flexible photocatalytic reactors, self-cleaning surfaces or wearable antimicrobial fabrics). Therefore, photochemical solution deposition (PCSD) demonstrates to be not only an efficient approach for the low temperature processing of oxide films, but also an excellent alternative for the stabilization of metastable phases.

The demand of energy-efficient fabrication processes and the need of integration of high-performance materials in the emerging flexible electronics have pushed the development of low temperature processing methods that allows obtaining functional oxide thin films at low temperatures^1^,^2^. Special attention has been paid to chemical solution deposition (CSD), because it offers benefits such as low cost, compositional control, large-area deposition or high-throughput fabrication. Additionally, CSD provides a particular advantage; the tailoring of the solution chemistry to achieve special processing targets (e.g., to reduce the high temperatures conventionally used, over 600 °C)^1^,^2^,^3^,^4^,^5^,^6^. Recent developments in the preparation of metal oxide thin films by solution methods have made use of a specifically design of homo- and hetero-metallic molecular complexes aimed either at the reproduction of a molecular structure similar to that of the crystal structure of the oxide phase to be synthesized in the film, or at the increase of their photosensitivity for irradiation purposes^2^,^7^,^8^,^9^,^10^,^11^. Within the framework of the CSD methodology, sensitive metal coordination complexes have shown a huge potential for the processing of oxide thin films at low temperatures (<400 °C), like several Pb- and Bi-based ferroelectric perovskites^10^,^11^. These compounds are characterized by reactive metal – to – ligand charge transfer states, where a shift of the electronic distribution can be induced by UV light, resulting in the dissociation of the complex bonds and the formation of the metal – O – metal skeleton of the oxide material^2^,^3^,^6^,^10^,^11^,^12^. As a result, photochemistry has become a powerful tool for the fabrication of films of crystalline oxides at low temperatures, minimizing the high energy consume of the traditional thermal annealing methods and providing a new pathway to integrate functional oxide layers with flexible polymers^2^,^12^.

However, we should consider that some important functional oxides present a variety of phases that are not thermodynamically stable at room temperature, but whose properties make them attractive for their integration in flexible devices. The use of tailored photosensitive solutions combined with PCSD methods seems to be the answer to this challenge: the stabilization of selected oxide phases far from their equilibrium conditions, using processing temperatures that allow their integration in real devices. We chose as a case study bismuth oxide. Besides the large interest that functional oxides containing elements with low levels of toxicity such as Bi have attracted, the single Bi_2_O_3_ oxides of Bi(III) present several polymorphic forms with excellent properties for applications^13^. Among them, the monoclinic α-Bi_2_O_3_ is stable at low temperatures up to 730 °C, while the cubic δ-Bi_2_O_3_ is the phase present at high temperatures (up to melting point at 824 °C). The rest of polymorphs are metastable phases at different temperature ranges, like the tetragonal β-Bi_2_O_3_ polymorph (450 °C–667 °C). It shows an excellent photocatalytic activity under visible light^4^,^13^. The introduction of impurities is a common strategy to stabilize this phase, but it leads to a strong decrease in the photoactivity. Other strategies have been used for the synthesis of this phase with different degrees of success^4^,^14^,^15^,^16^,^17^,^18^. Most of them have focused on the preparation of pure β-Bi_2_O_3_ nanostructure materials (nanopowders, nanoflakes, nanowires or nanosheets), by different approaches such as induced aqueous synthesis^19^, formation of bismuth oxido clusters precursors^15^,^20^, metal vapor transport, induced template^4^, hydrothermal methods^21^,^22^ or microwave processes^22^. However, for the practical use of the photocatalyst material, the β-Bi_2_O_3_ powder needs to be dispersed in the wastewater for purification and then removed. This may lead to secondary pollution of the water by residual photocatalyst, and increases the cost of the process. This problem can be circumvented by supporting the catalyst on a substrate. Among the scarce works on pure β-Bi_2_O_3_ thin films, note that these are usually obtained on traditional substrates by deposition techniques that involve a thermal treatment at high temperature^23^,^24^,^25^,^26^. The unavailability of a method to prepare β-Bi_2_O_3_ films at sufficiently low temperatures hampers the use of cheap and flexible plastic substrates for photocatalytic reactors. However, flexible photocatalysts films would enlarge the application of these materials to other areas such as self-cleaning surfaces or wearable antimicrobial clothes, at present not possible^27^,^28^.

In this work, we show that the selection of a metal complex precursor with a molecular structure close to that of β-Bi_2_O_3_ allows the preparation of thin films of this metastable phase at temperatures far below its equilibrium conditions by PhotoChemical Solution Deposition (PCSD) methods. We also demonstrate that the phase is stable for a wide temperature range, starting at room temperature. Besides a systematic structural analysis of the films and a study of their conductivity behavior in order to clearly identify de β-Bi_2_O_3_ among all polymorphs^14^,^29^, the demonstration of the photocatalytic ability of the films shows their potential for applications. The implications of the successful application of the PCSD methods to obtain films of metastable metal oxides on flexible substrates are discussed.

## Results and Discussion

**Figure 1:**
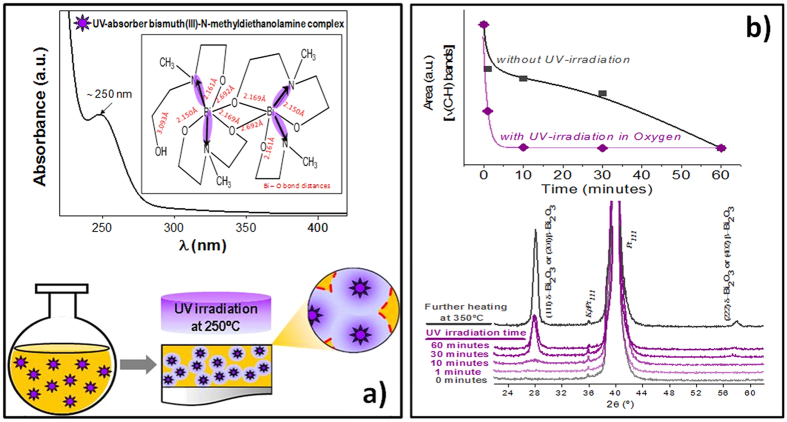
Photosynthesis of β-Bi_2_O_3_ thin films. (**a**) Scheme of the photochemical process of preparation of crystalline Bi_2_O_3_ thin films from UV-absorbing Bi(III) solutions containing a Bi(III)-*N*-methyldiethanolamine coordination complex with a maximum of light absorption at λ ~ 250 nm (synthesis of solutions is shown in Figure S2). (**b**) The photoreaction produced in the gel layers derived from the former precursor solutions by the irradiation with UV-light is followed by the decrease of the integrated area of the ν(C–H) stretching vibrations ascribed to the C-H groups (ν(C–H)_O_ ~ 2960 nm, ν(C–H)_CO_ ~ 2900 nm and ν(C–H)_N_ ~ 2860 nm), as a function of the irradiation time at 250 °C in an oxygen atmosphere (graphic at the top). The evolution of the crystallinity of the former layers with the irradiation time at 250 °C, and with a further treatment at 350 °C was followed by X-ray diffraction (graphic at the bottom).

The photosensitive metal complex of Bi(III) and *N*-methyldiethanolamine shown in Fig. 1a was synthesized in solution as described elsewhere, Figure S1^11^. This UV-absorbing Bi(III) solution was used for the deposition of amorphous layers on substrates that were UV-irradiated in oxygen at a temperature of 250 °C. The study of the photoreaction produced in these layers indicates that UV-light induces the prompt elimination of the organics (irradiation for only 10 minutes at 250 °C). As the irradiation time increases, Bi_2_O_3_ crystalline films appear (Fig. 1b). Crystallinity of these films is improved with the increase of the temperature and the oxidant effect of the irradiation atmosphere (Figures S2).

**Figure 2:**
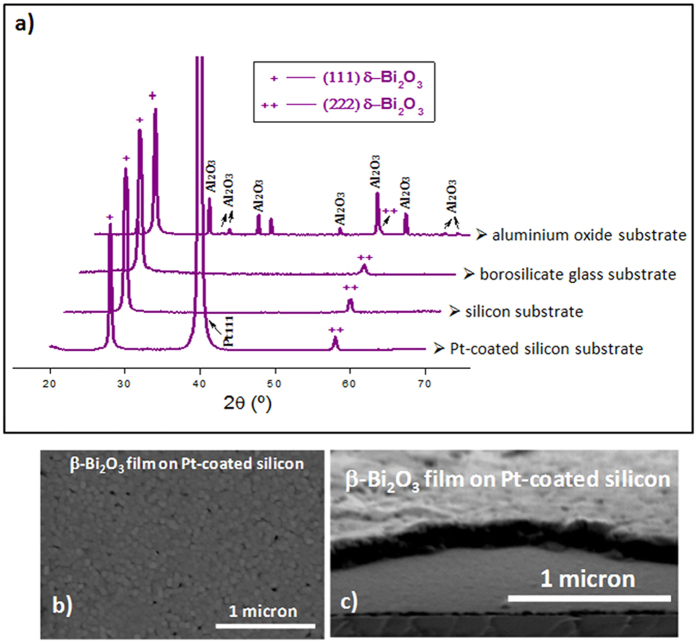
Bi_2_O_3_ thin films on different substrates. (**a**) X-ray diffraction (XRD) patterns of ~45 nm thick Bi_2_O_3_ thin films fabricated on different substrates from UV-absorbing Bi(III) gel layers heated at 250 °C in an oxygen atmosphere with UV-irradiation and subjected to a further rapid thermal annealing in oxygen at 350 °C. Field emission gun scanning electron (FEGSEM) microscopy photographs of β-Bi_2_O_3_ thin films on a Pt-coated silicon substrate prepared from the UV-absorbing Bi(III) precursors with UV-irradiation: (**b**) surface image (**c)** cross-section image.

The crystalline structures of the films formed after this UV-assisted low temperature processing are analyzed by X-ray diffraction (XRD), with a conventional Bragg-Brentano geometry (Fig. 2a). The peaks of the diffraction patterns can be assigned to the reflections recorded in those patterns reported for β-Bi_2_O_3_ powders and films^15^,^19^,^23^, but also to those of the published X-ray diffractograms for the high-temperature δ-Bi_2_O_3_ films stabilized at room temperature with a <111> preferred orientation^30^,^31^. It must be noted that when UV-irradiation is not used and/or non-UV-absorbing Bi(III) gel layers are deposited, the crystallization at such low temperature leads to an undesirable mixture of Bi_2_O_3_ phases (Figure S3). Additionally, it is noteworthy that the formation and stabilization of the isolated polymorph is substrate independent; it is formed on (111) Pt – coated (100) silicon, SiO_2_ – (100) silicon, amorphous borosilicate glass substrates or polycrystalline corundum substrates (Fig. 2b,c and Figure S4). Besides, films with nanometer grain sizes are always obtained on the different substrates. Even, the bad wetting of the borosilicate glass substrate by the film does not seem to restrict the formation of this Bi_2_O_3_ phase also in this material (Figure S4h).

**Figure 3:**
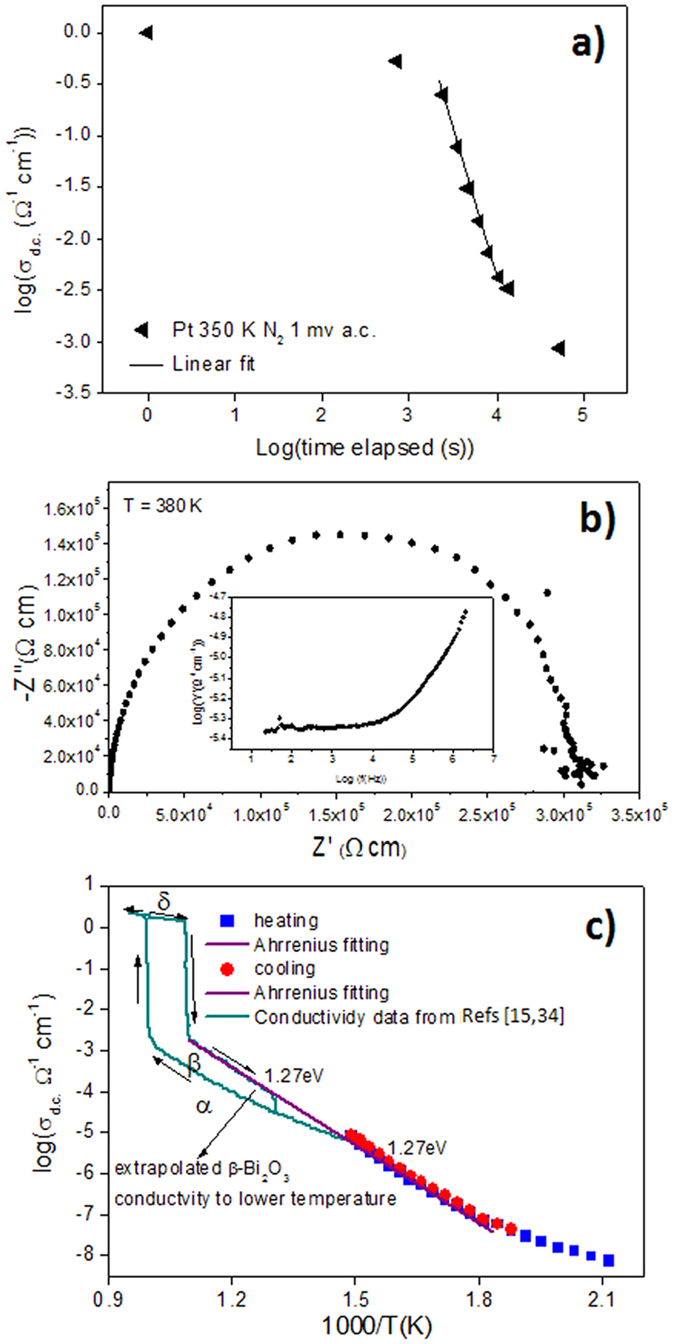
Conductivity behaviour of β-Bi_2_O_3_ thin films. (**a)** Conductivity as a function of time of a ~45 nm thick Bi_2_O_3_ thin film on a (111)Pt/TiO_2_/SiO_2_/(100)Si substrate. Measurements were carried out on planar capacitors fabricated by the deposition on the film surface of ~200 μm diameter Au electrodes. (**b**) Impedance diagram measured at 380 K for a ~190 nm thick Bi_2_O_3_ thin film on a Al_2_O_3_ substrate with interdigital Au electrodes (IDE) deposited on the film surface (Figure S5). The lack of Au electrode blocking at low frequencies (inset) indicates a dominant electronic conductivity. (**c**) Conductivity as a function of temperature in the interval of 460–680 K of the former Bi_2_O_3_ thin film.

Conductivity measurements were carried out on these films looking for the very different conductivity behavior of the β-Bi_2_O_3_ and δ-Bi_2_O_3_ polymorphs that would allow us differentiate which of the two phases has been stabilized in the former films. An electronic conductivity is expected for the β-Bi_2_O_3_, whereas the δ-Bi_2_O_3_ has the highest oxide-ion conductivity of all of the binary metal oxides^13^,^14^,^32^. The conductivity of these films initially measured on planar capacitors shows a chaotic behavior similar to that reported for δ-Bi_2_O_3_ films (Fig. 3a)^33^,^34^, i.e., a strong decrease of resistivity is obtained after the application of high electric fields. This phenomenon was attributed to the formation of metallic Bi nanofilaments in Bi_2_O_3_ films between the top and bottom electrode during the application of high fields^34^. But, a much more careful measurement of the conductivity could be carried out by using capacitors with interdigital electrodes on the surface of the films deposited on the Al_2_O_3_ substrate (see experimental set-up in Figure S5). The impedance diagram measured at 380 K for this film is shown in Fig. 3b. The lack of Au electrode blocking for low frequencies (see inset) indicates a dominant electronic conductivity without the prevalence of ion conductivity. Furthermore, the variation of the conductivity of this film with temperature (Fig. 3c) measured, to the knowledge of the authors, for the first time at temperatures close to room temperature shows a behavior that agrees with that obtained for bulk materials at the high temperatures where the β-Bi_2_O_3_ polymorph is stable^14^,^35^.

**Figure 4:**
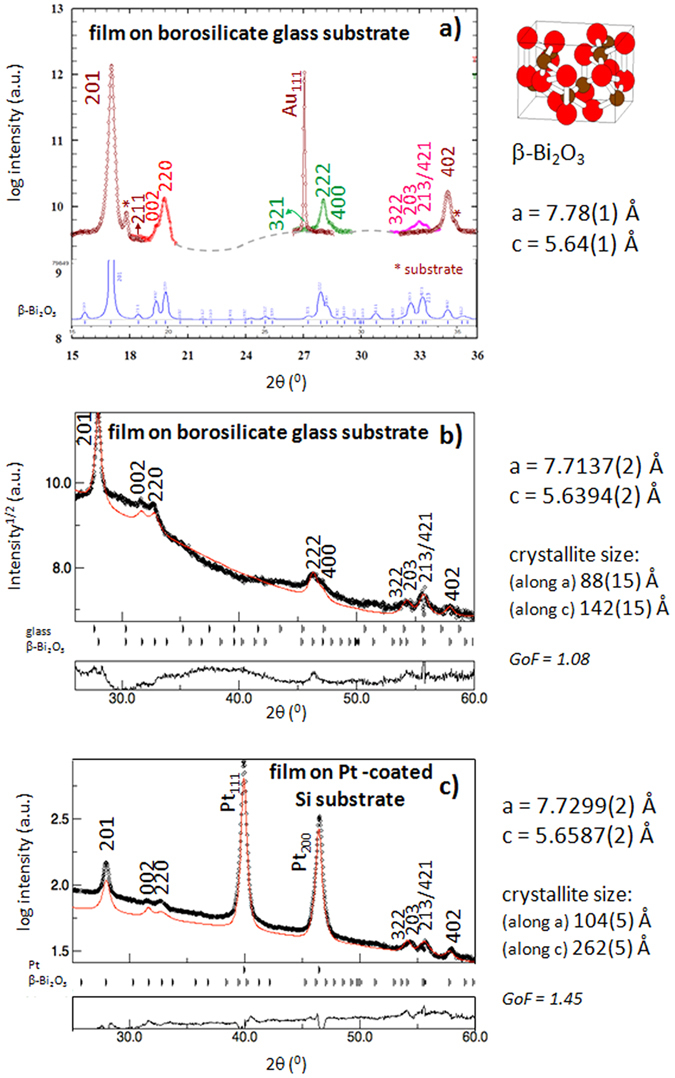
Crystal structure of β-Bi_2_O_3_ thin films. (**a**) X-ray diffraction pattern of a Bi_2_O_3_ thin film deposited on a borosilicate glass substrate (synchrotron radiation, λ = 0.95 Å). Measurements were carried out at different sample angles in order to bring into diffraction the maximum number of planes of the film. The results are superimposed in a unique pattern and identified with different colors. A first estimation of its lattice parameters is shown. Diagram at the bottom correspond to the pattern of the β-Bi_2_O_3_ phase calculated with the PowerCell program^34^. (**b**) Sum of XRD patterns (Cu anode, λ = 1.5406 Å) obtained at different sample orientations (grid of 5° × 5° in χ and φ) for the same Bi_2_O_3_ thin film on a borosilicate glass substrate and (**c**) for another film deposited on a Pt-coated Si substrate. Rietveld refinements (red solid line) reveals that the best fitting is obtained for the tetragonal β-Bi_2_O_3_ polymorph. Cell parameters and crystallite sizes are calculated for both films. Diagrams at the bottom of (**b** and **c**) correspond to the difference between the experimental and calculated patterns. The goodness of fit (GoF) is included.

Therefore, the conductivity behavior indicates that the β-Bi_2_O_3_ polymorph is the phase stabilized in these films, and not the cubic δ-Bi_2_O_3_ crystal phase. However, an accurate identification of the crystal structure cannot be made with the two only reflections of the phase recorded in the XRD patterns of Fig. 2a. In order to determine the true nature of the polymorph obtained, high resolution X-ray diffraction experiments using synchrotron radiation have been carried out on one of the films deposited onto a borosilicate glass substrate. As the prevailing texture practically extinguishes the majority of the observable diffraction peaks in a symmetric θ–2θ experiment, an on purpose sequence of film orientations and measurements was performed. The results obtained for different ad hoc orientations of the film are collected in Figure S6. Figure 4a shows the results of a conveniently weighted superposition of measurements. Instead of the single peaks characteristic of the cubic fluorite δ-Bi_2_O_3_ phase, broad and asymmetric peaks are indexed with respect to the tetragonal β-Bi_2_O_3_ polymorph (detail of the peaks in Figure S6c). A good agreement is observed between the experimental and the calculated patterns^36^. A first estimation of the cell parameters of the β-Bi_2_O_3_ phase is obtained.

This study was complemented with the analysis of the XRD data obtained for a systematic rotation of the sample (grid of 5° × 5° in χ and φ) with a four-circle goniometer. Besides the determination of the crystallographic texture of the films (Figure S7), the Combined Analysis refinement^37^,^38^ carried out confirms the stabilization of the metastable β-Bi_2_O_3_ phase in the film. Figure 4b,c show the experimental sum and the fitted patterns for two films deposited on borosilicate glass and on Pt-coated silicon substrates. A good agreement is obtained using the P
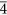
2_1_c space group corresponding to tetragonal β-Bi_2_O_3_ [Crystallography Open Database no. 9007723]^39^, whereas the refinement with Fm
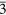
m of the cubic δ-Bi_2_O_3_ [COD no. 1534843] leads to poor reliability factors. The lattice parameters and the crystallite sizes calculated for both β-Bi_2_O_3_ films are indicated in the figure. In spite of the very different nature of the substrate, texture is similar in both films (Figure S7). Crystallite sizes are smaller for the film on glass, which is in agreement with the smaller grain size observed for this film in the scanning electron microscopy images of Figure S4g,h.

Residual stresses were calculated for two films deposited on Pt-coated silicon substrates (Figure S8, Table S1). The results indicate that the UV-irradiated film with the desired β-Bi_2_O_3_ phase is stress free (typically less than few MPa), unlike the film prepared without irradiation, which calculated residual stresses are of the order of 10 GPa. Brillouin measurements carried out on these films confirm these results (Figure S9). Differences in the elastic properties of the substrate, where each of the films is supported, are obtained. This indicates different mechanical stresses generated by the preparation of the metastable β-Bi_2_O_3_ film or the Bi_2_O_3_ film. Again, no significant stresses are measured in the β-Bi_2_O_3_ film, since the propagation of the surface acoustic wave in the substrate to which it is attached is the same as that of the substrate without any coating. Also, no visible residual strain could be seen in the XRD Combined Analysis, which indicates that no stress is stabilized in the film larger than 10–20 MPa in our experimental resolution (see Figure S7a). On the contrary, stresses are developed in the Bi_2_O_3_ film’s substrate without UV irradiation.

The absence of residual stress in the irradiated film contrasts with the increase of the bulk density in this film, which is around a 10% less thick than the film without irradiation. This is a typical effect produced by the exposure of sol-gel layers to UV light^3^. The shrinkage produced during crystallization is therefore larger in the irradiated films. This should result in a large residual stress in the final film, contrary to the fact occurring here.

It is proven that the thin film conformation can force the stabilization of non-equilibrium phases by e.g., lattice mismatch accommodation between film and substrate, strain-induced structure engineering, imprisonment of the crystal structure in the nanometer range or design of specific heterostructures^30^,^31^,^40^. However, this seems not to be the case here since our results prove that the formation and stabilization of the β-Bi_2_O_3_ phase does not depend on the substrate effect, neither on the nanometer nature of the film (Fig. 2 and Figure S4).

The molecular structure of the bismuth (III) – *N*-methyldiethanolamine coordination complex has been determined by single crystal X-ray diffraction^8^, indicating that bismuth atoms are surrounded by three short primary Bi – O bonds (2.150 Å), two transannular Bi – N bonds (2.729 Å, 2–746 Å) and two long Bi – O bonds corresponding to the association of the monomeric units into dimmers with asymmetrical Bi – O bridges (2.169 Å, 2.692 Å), as well as to the partially Bi – O(H) deprotonated diol (3.093 Å) (inset of Fig. 1a). With the exception of the Bi – O(H) bond, these distances are in the range of those measured for the β-Bi_2_O_3_ polymorph, with the bismuth in a pseudo-octahedral geometry^8^,^41^,^42^,^43^,^44^. Therefore, the transition from one to the other seems natural and can occur at very low temperature, as it happens here.

Taking into account the molecular structure of the photosensitive precursor and the effect of the UV-irradiation on the layers deposited from it, we may hypothesize a possible mechanism of formation and stabilization of these β-Bi_2_O_3_ thin films. Initially, the drying of the deposited layers containing the Bi(III)–*N*-methyldiethanolamine complex induces the formation of an amorphous – Bi – O – Bi – network with interatomic distances close to those of the tetragonal crystal structure of the β-Bi_2_O_3_ polymorph. Therefore, the formation of β-Bi_2_O_3_ crystals is easy in these films at a low temperature (250 °C) (Figure S3c). The further UV irradiation accelerates crystallization^2^,^3^,^10^,^12^,^45^ in a matrix that is dense enough to allow the effective coalescence of the formed crystallites (Table S1). These crystallites will work like seeds for the full crystallization of the tetragonal β-Bi_2_O_3_ phase as the irradiation time at 250 °C or the annealing temperature is increased (Figs 1b and S3d), under favorable not-constrained boundary conditions. Thus, the resulting β-Bi_2_O_3_ films are not subjected neither residual strain nor stresses (Table S1, Figures S7a and S9). This could be explained by the straightforward conversion from the amorphous – Bi – O – Bi – O – cross-linking network to the tetragonal β-Bi_2_O_3_ crystal phase, both with close Bi – O interatomic distances, and besides leading to a film with small grains that are able of absorbing efficiently the deformation of the substrate and relaxing stresses through the film thickness. Furthermore, not only this metastable β-Bi_2_O_3_ phase is stabilized at room temperature in these films processed at a temperature of only 250 °C, far from the formation and stabilization temperature of the polymorph, but also the films show a wide temperature stability range, between room temperature and 450 °C (Figure S10), which is extremely important for the applications of these materials. In the case of the non-irradiated films, at this first stage of drying and treatment at 250 °C, the β-Bi_2_O_3_ nano-crystallites formed are surrounded by an amorphous and porous matrix (Figure S4c). Since these initial crystallites cannot interact so closely like in the irradiated films, due to the porous amorphous matrix, they do not longer rule the growth of the β-phase. Therefore, an increase of the heating temperature up to 350 °C should naturally induce the conversion of this amorphous matrix into the stable bismuth oxide phase at this temperature, which is the α-Bi_2_O_3_ phase, with a larger grain size than that of the irradiated films (Figures S3c,d and S4), hindering the stress relaxation (Table S1, Figures S7a and S9).

**Figure 5:**
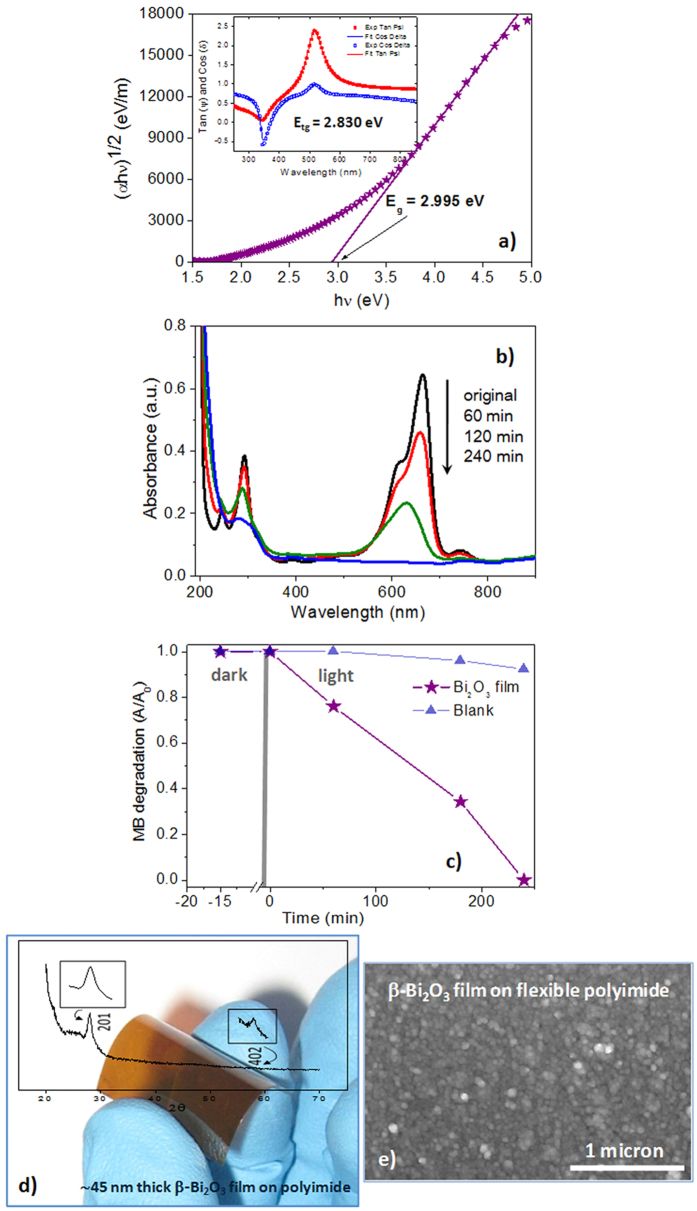
Functionality of the β-Bi_2_O_3_ thin films. (**a**) Spectral dependence of the absorption coefficient, α, as a function of the photon energy. Optical properties (n(λ), k(λ) and α) were calculated from the data recorded by spectroscopic ellipsometry (inset of a). (**b**) photocatalytic degradation of methylene blue (MB) by a β-Bi_2_O_3_ thin film on Pt-coated Si substrate. The film thickness was ~50 nm and the area of ~2 cm^2^. (**c**) Photocatalytic degradation kinetics of methylene blue (MB) over the former film. (**d**) Photograph of a ~45 nm thick β-Bi_2_O_3_ on flexible polyimide. The inset shows the X-ray pattern of this film measured with a conventional Bragg-Brentano geometry in a Brucker powder diffractometer with a Cu anode (λ = 1.5406 Å). (**e**) SEM surface image of the β-Bi_2_O_3_ on the flexible polyimide substrate.

Optical characteristics of the β-Bi_2_O_3_ thin films are shown in Fig. 5a. An indirect and a Tauc band gap of E_g_ ~ 2.995 eV and E_tg_ ~ 2.830 eV are calculated from these results, respectively. This means that these β-Bi_2_O_3_ films show light absorption in the visible range (Figure S11a). Taking into account these characteristics and the potential of β-Bi_2_O_3_ materials for the photocatalytic degradation of dyes, photocatalytic experiments were carried out using methylene blue, MB, as pollulant. Figure 5b and c shows the photocatalytic degradation of MB over a β-Bi_2_O_3_ thin film with an area of ~2 cm^2^ and a thickness of ~50 nm (Figure S11b). The film shows to be efficient for the degradation of the dye, which is totally degraded for short times of light exposure. Therefore, these results demonstrate the efficiency of these thin film materials for visible-light photocatalysis^4^,^15^,^18^,^46^,^47^,^48^ that should be associated to the high purity of the β-Bi_2_O_3_ polymorph achieved by this synthesis strategy.

But, a main feature of this work is to make available the fabrication of these β-Bi_2_O_3_ thin films on low-melting point substrates, such as flexible polyimide (Fig. 5d). Hence, this photochemical method provides a new pathway to prepare the metastable β-Bi_2_O_3_ polymorph at a very low temperature (250 °C) far from the temperatures at which the phase is thermodynamically stable (450–667 °C). Specifically, this is an important added value for this material, since it makes real the integration of β-Bi_2_O_3_ thin films with flexible polymers, enlarging the applications of the devices prepared with this compound (e.g., adaptable flexible light-weight water pipeline photocatalytic reactors, self-cleaning surfaces, wearable antimicrobial fabrics)^27^,^28^. But, from a general point of view, an adequate design of photosensitive metal complexes combined with the use of PCSD can open the window for the preparation of films of other metastable phases of interest for the upcoming flexible devices.

## Concluding Remarks

The results of this work demonstrate that Photochemical Solution Deposition (PCSD) methods can be used, with appropriate tailored precursors, to fabricate films of non-equilibrium crystalline phases of functional metal oxides. This processing technology is successfully applied to the preparation of thin films of the metastable β-Bi_2_O_3_ phase from strong UV-absorbing precursors at only 250 °C, far from the formation temperature of this polymorph, ~650 °C. The opportunity for the fabrication of these films at so low temperature makes possible their integration with flexible plastic substrates, extending the potentiality of these β-Bi_2_O_3_ thin films for applications. Besides, the metastable phase is stable at room temperature, showing a wide temperature stability range up to 450 °C, thus enlarging the working operation range of these materials. Therefore, this photochemical solution synthesis method reveals itself as a powerful strategy to design and stabilize metastable crystalline oxide phases at room temperature, thus enabling the access to their promising properties at optimal conditions and anticipating unexplored applications until now.

## Materials and Methods

Bi_2_O_3_ thin films on different substrates were obtained by the spin-coating deposition of precursor solutions of Bi(III) prepared with and without *N*-methyldiethanolamine (CH_3_N(CH_2_CH_2_OH)_2_. The layers were dried at 150 °C for 10 min and heated at 250 °C in an O_2_ atmosphere, with or without UV-irradiation. For the UV-irradiation, an excimer lamp (Heraeus-Noblelight Bluelight Curing Module) with λ_emission_ = 222 nm, electrical power of 1.5 kW, frequency of 50 Hz, irradiation length of 30 cm and irradiance of 6.25 W.cm^−2^ was used. Samples were placed in a closed chamber, containing a furnace for heating with a distance of the sample to the UV-lamp of 9 cm. Further heatings of the samples were carried out in a rapid thermal processor (RTP, JetStar 100 JIPELEC equipment) (Figure S1). The crystallinity of the films was followed by X-ray diffraction, using a Siemens D500 powder diffractometer with a Cu anode (λ = 1.5406 Å) and a Bragg-Brentano geometry. JCPDS-ICDD files were used to identify the bismuth oxide phases (α-Bi_2_O_3_  →  JCPDS-ICDD-41-1449, β-Bi_2_O_3_ → JCPDS-ICDD-27-0050, δ-Bi_2_O_3_  →  JCPDS-ICDD-27-0052, γ-Bi_2_O_3_  →  polymorph phase reported by Harwig *et al*.^35^, ε-Bi_2_O_3_ → polymorph phase reported by Corne *et al*. and Locherer *et al*.^44^,^49^. Bi_2_O_3_ silenite → JCPDS-ICDD-74-2351, non-stoichiometric Bi_2_O_2.33_ → JCPDS-ICDD-27-0049, Bi_2_O_2.75_ → JCPDS-ICDD-27-0051 or Bi → JCPDS-ICDD-05-0519). Pt peaks are adjusted to the JCPDS-ICDD file 4-0802. Peaks from the Al_2_O_3_ substrate are adjusted to the JCPDS-ICDD-42-1468 file (corundum). X-ray diffraction patterns of the films were also obtained by synchrotron radiation with a wavelength of λ = 0.95 Å in the beamline MCX of the Elettra-Sincrotrone Trieste (Figure S6). The theoretical pattern for the β-Bi_2_O_3_ phase was calculated using the software of ref. 36. Additional X-ray measurements were carried out in a diffractometer equipped with a four-circle opened Eulerian goniometer (χ, φ), a Cu anode (λ = 1.5406 Å), a 120° curved linear position-sensitive detector (CPS120 from INEL SA) and a flat graphite primary monochromator. Combined analysis (texture, microstructure using an extended Rietveld refinements) of the XRD data were carried out using the Materials Analysis Using Diffraction package (MAUD)^37^. Cross-section and plan-view micrographs of the crystalline oxide films were obtained by field-emission gun scanning electron microscopy (FEG-SEM, Nova Nanosem 230 FEI Company equipment, Hillsboro, OR). Conductivity as a function of temperature of β-Bi_2_O_3_ thin films on Al_2_O_3_ and Pt-coated Si substrates were carried out on films with interdigital or planar electrodes (~190 nm and 45 nm thick films on Al_2_O_3_ and Pt-Si, respectively). Measurements were carried out on a heating and cooling cycle in the temperature interval between room temperature and 400 °C, increasing temperature in 20 °C steps. For these measurements 48 pairs of IDE Au electrodes of 20 μm of thickness, 1400 μm of length and a separation between them of 20 μm, were deposited on the film on Al_2_O_3_ substrate. Planar capacitors were fabricated with the films on Pt-Si, by the deposition of ~200 μm diameter Au electrodes, by sputtering using a shadow mask. Stresses developed in Bi_2_O_3_ and β-Bi_2_O_3_ films on Pt-coated silicon substrates were obtained from the curvature radii calculated by profilometry (Figure S8 and Table SI). Film thickness and optical properties were obtained by spectroscopic ellipsometry (SOPRA GES5E ellipsometer at the IR-Spectroscopy service of the ICMM-CSIC) in the wavelength range from 200 nm to 850 nm. Data were analyzed with the Winelli II software, using a Tauc-Lorentz model. The experimental set up for High Resolution Surface Brillouin Spectroscopy (HRSBS) is described elsewhere^50^. The light source was a 2060 Beamlok Spectra Physics Ar^+^ ion laser provided with an intracavity temperature stabilized single-mode and single-frequency z-lok etalon (λ_0_ = 514.5 nm). The scattered light was analyzed using a Sandercock-type 3 + 3 tandem Fabry-Pérot interferometer. Values for Finesse and contrast were 150 and 10^9^, respectively. To couple the incident light beam to the surface phonons, the incident beam is polarized in the scattering plane and the related scattering wave vector, 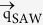, is parallel to the sample surface. The SAW propagation velocity is obtained from the surface phonon frequency f_SAW_:


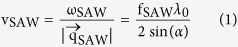


where λ_0_ is the laser wavelength in vacuum and α is the scattering angle or sagittal angle.

The photocatalytic activity of the β-Bi_2_O_3_ films was evaluated by the degradation of methylene blue, MB (C_16_H_18_ClN_3_S.xH_2_O). A β-Bi_2_O_3_ film with ~2 cm^2^ of area and a thickness of ~50 nm was placed inside a chamber with 8 mL of the dye solution (10^−6^ mol/L) and with a pH ~ 1, fixed with hydrochloric acid (HCl). The Photocatalytic reactor was equipped with a solar lamp (Ultra-Vitalux 300 W, Osram) emitting in UVA (13.6 W) and UVB (3.0 W) regions and using a borosilicate window as a light filter. The temperature in the reactor was kept below 38 °C by a cooling system. Prior to irradiation, the suspension was in the dark for 15 min to reach an adsorption/desorption equilibrium.

## Additional Information

**How to cite this article**: Pérez-Mezcua, D. *et al*. Photochemical solution processing of films of metastable phases for flexible devices: the β-Bi_2_O_3_ polymorph. *Sci. Rep.* **6**, 39561; doi: 10.1038/srep39561 (2016).

**Publisher's note:** Springer Nature remains neutral with regard to jurisdictional claims in published maps and institutional affiliations.

## Supplementary Material

Supplementary Information
