# Supplementary material for: Photochemical solution processing of films of metastable phases for flexible devices: the β-Bi2O3 polymorph
Source: Sci Rep. 2016 Dec 20;6:39561. doi: 10.1038/srep39561 (PMC5171705; doi:10.1038/srep39561)
Supplement: Supplementary Information [file srep39561-s1.pdf]

Supplementary Information

Photochemical solution processing of films of metastable phases for flexible devices: the β-Bi_2O_3 polymorph

*Dulce Pérez-Mezcua[♣,ψ], Iñigo Bretos[♣], Ricardo Jiménez[♣], Jesús Ricote[♣], Rafael J. Jiménez-Rioboó[♣], Cosmelina Gonçalves da Silva, Daniel Chateigner[&], Luis Fuentes[ϒ], Rafael Sirera[ψ] and M. Lourdes Calzada[♣]**

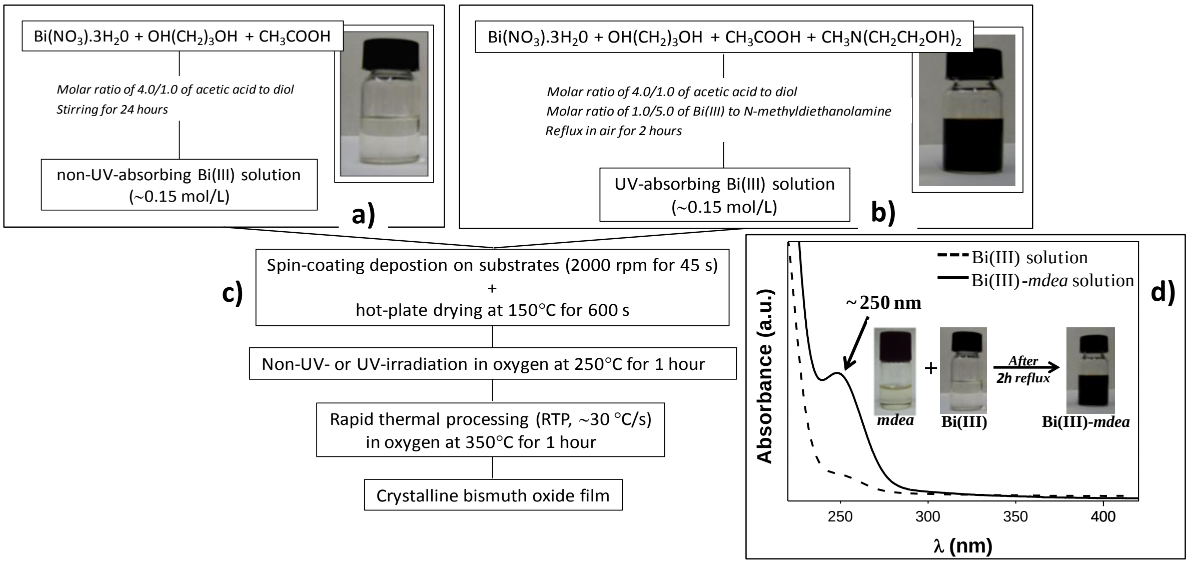

Figure S1. **Preparation of Bi_2O_3 thin films**. **a)** and **b)** Schemes for the preparation of the Bi(III) precursor solutions: non-UV-absorbing Bi(III) solution and UV-absorbing Bi(III) solution. The non-UV absorbing Bi(III) solution (~0.15 equivalent moles of Bi(III) per liter of solution) was obtained by mixing bismuth (III) nitrate hydrated ($Bi(NO_3)_3{\cdot}5H_2O$, Aldrich, 99.99%) in 1,3-propanediol ($HO(CH_2)3OH$, Aldrich, 98%) and acetic acid (CH_3COOH, Merck, 100%) and stirring for 24 hours in air, using a 1.0/4.0 propanediol / acetic acid molar ratio. A transparent solution was obtained, here called Bi(III) solution. The UV-absorbing Bi(III) solution (~0.15 equivalent moles of Bi(III) per liter of solution) was prepared by refluxing for 1 hour in air bismuth (III) nitrate hydrated with N-methyldiethanolamine ($CH_3N(CH_2CH_2OH)_2$, Aldrich, 99%) (*mdea*), in 1,3-propanediol and acetic acid solvents (1.0/4.0 diol / acetic acid and 1.0/5.0 Bi(III) / $CH_3N(CH_2CH_2OH)_2$ molar ratios). A reddish-brown colored sol was obtained, here called Bi(III)-*mdea* solution. **c)** Preparation of bismuth oxide thin films. For UV-irradiation, an excimer lamp (heraeus-Noblelight Bluelight Curing Module) with λemission=222nm, an electrical power of 1.5 kW, a frequency of 50 Hz, an irradiation length of 30 cm and an optical irradiance of 6.25 W/cm^2 was used. Samples were placed in a closed chamber, containing a furnace for the heating of the samples. This chamber has a top quartz window transparent to the UV-light. The distance of the sample to the UV-lamp is 9 cm.[1] (2) Crystallization of the films was carried out in a rapid thermal processor (RTP, JetStar 100 JIPELEC equipment) at 350°C for 1 hour in an oxygen atmosphere and with a heating rate of ~30°C/s). **d)** UV spectra of the Bi(III) and Bi(III)–*mdea* solutions, showing a maximum of absorbance for the Bi(III)-*mdea* solution at ~250 nm.[2]

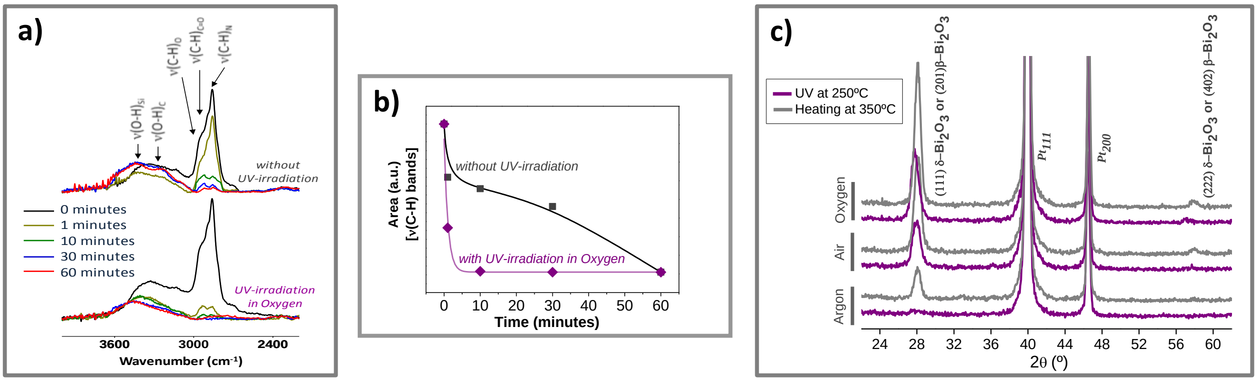

Figure S2. **Photochemistry of the UV-absorbing Bi(III) films**. **a)** Fourier transform infrared (FTIR) spectra of the films deposited from the UV-absorbing Bi(III) solution and treated at 250°C in O_2 atmosphere for 0, 1, 10, 30 and 60 minutes, without UV-irradiation and with UV-irradiation. The films were deposited on silicon doubled side polished substrates. **b)** Decrease of the integrated area of the ν(C–H) stretching vibrations as a function of the irradiation time. These bands are ascribed to three different C–H groups: the ν(C–H) at ~2960 cm^{-1} corresponds to C–H groups close to O- (denoted as $\nu(C–H)_O$), the ν(C–H) at ~2900 cm^{-1} of those C–H groups close to the carbonyl groups (denoted as $\nu(C–H)_{CO}$) and the ν(C–H) at ~2860 cm^{-1} of the C–H groups close to N- (denoted as $\nu(C–H)_N$).[2] **c)** X-ray diffraction (XRD) patterns the fil+ms deposited on (111)Pt/TiO_2/SiO_2/(100)Si substrates from the UV-absorbing Bi(III) solution and UV-irradiated for 60 minutes in argon, air or oxygen atmospheres during heating at 250°C and subsequently annealed in these atmospheres at at 350°C.

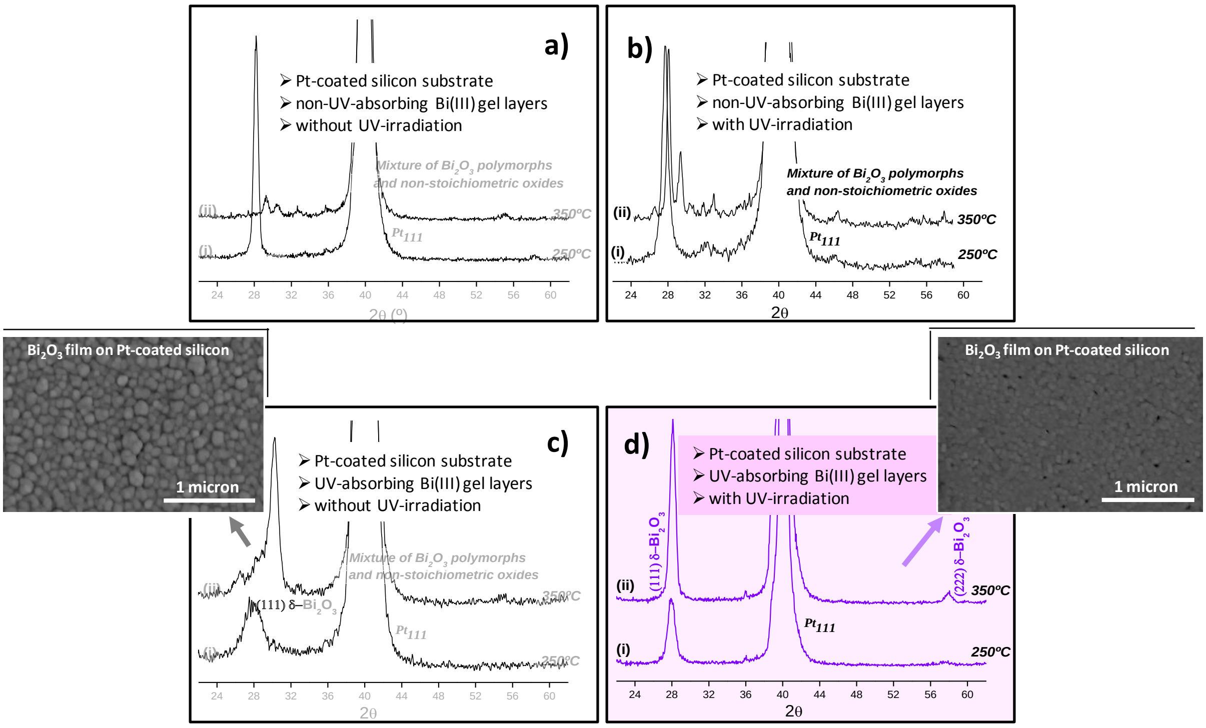

Figure S3. **Bi_2O_3 thin films**. **a)**, **b)**, **c)** and **d)** X-ray diffraction (XRD) patterns of ~ 45 nm thick Bi_2O_3 thin films fabricated on (111)Pt/TiO_2/SiO_2/(100)Si substrates from non-UV-absorbing and from UV-absorbing Bi(III) gel layers. (i) the deposited layers were heated for 1 hour at 250°C in an oxygen atmosphere, without UV-irradiation or with UV-irradiation. (ii) The former layers were subjected to a further rapid thermal annealing in oxygen at 350°C for 1 hour with a heating rate of 30°C/s. A Siemens D500 powder diffractometer with a Cu anode (λ=1.5406 Å) and a Bragg-Brentano geometry was used for the measurement of these XRD patterns. Insets of c) and d) correspond to the field emission gun scanning electron microscopy images of films surfaces obtained at 350°C.

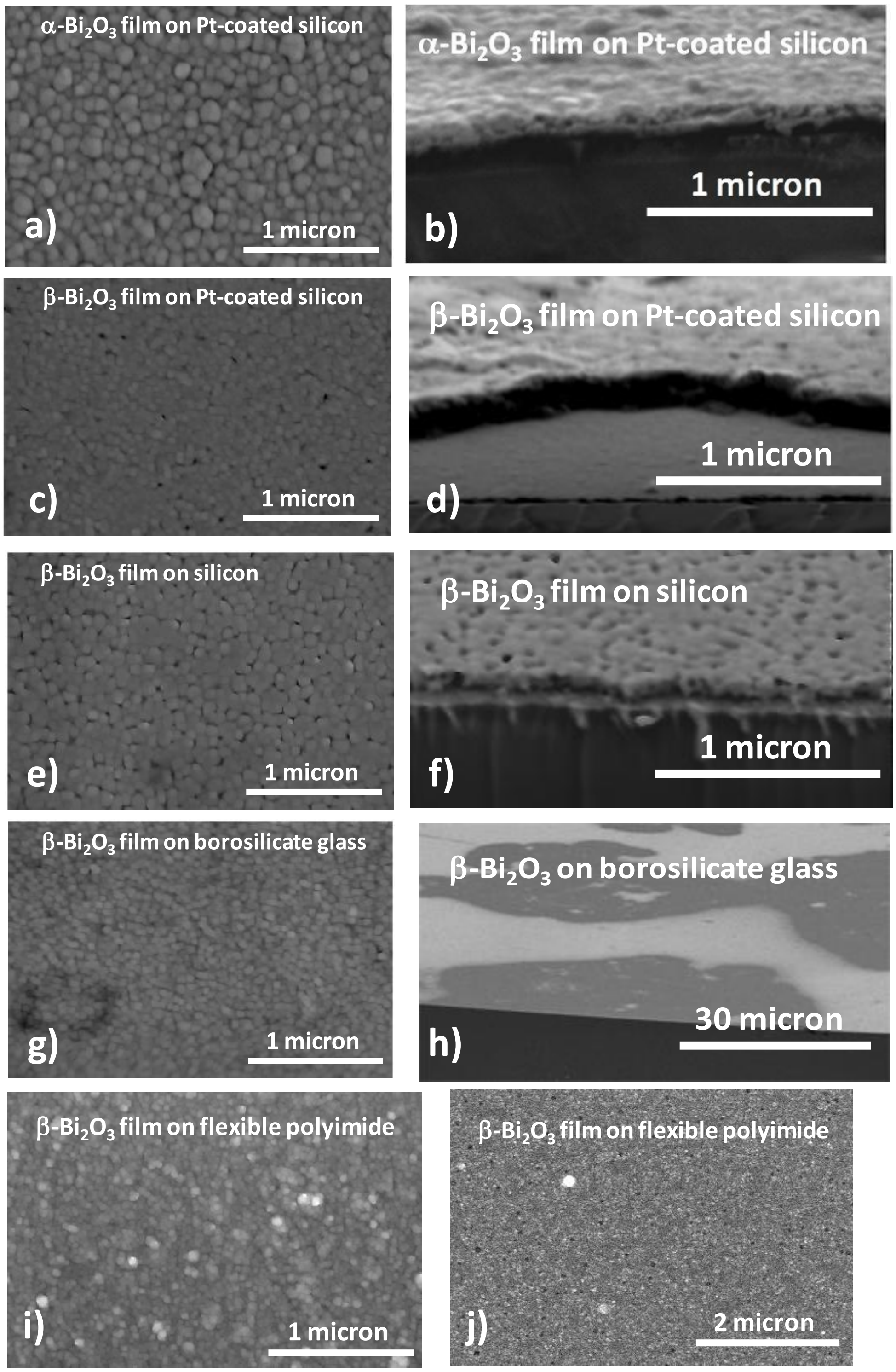

Figure S4. Field emission gun scanning electron microscopy photographs of Bi_2O_3 thin films on different substrates: a), b) surface and cross-section images of a α-Bi_2O_3 film on a (111)Pt/TiO_2/SiO_2/(100)Si substrate, c), d) surface and cross-section images of a β-Bi_2O_3 film on a (111)Pt/TiO_2/SiO_2/(100)Si substrate, e), f) surface and cross-section images of a β -Bi_2O_3 film on a SiO_2/(100)Si substrate and g), h) surface and cross-section images of a β -Bi_2O_3 film on an amorphous borosilicate glass substrate. h), i) surface images of a ~ 45 nm thick β-Bi_2O_3 film on a flexible polyimide substrate. Different magnifications are shown. The lower magnification show some defects in the film surface that the authors associate to the small thickness of the layer, the thermal decomposition of the Bi(III)-metal complex during UV-irradiation and/or a deficient wetting. Work is in progress to avoid these layer defects of the flexible β-Bi_2O_3 material.

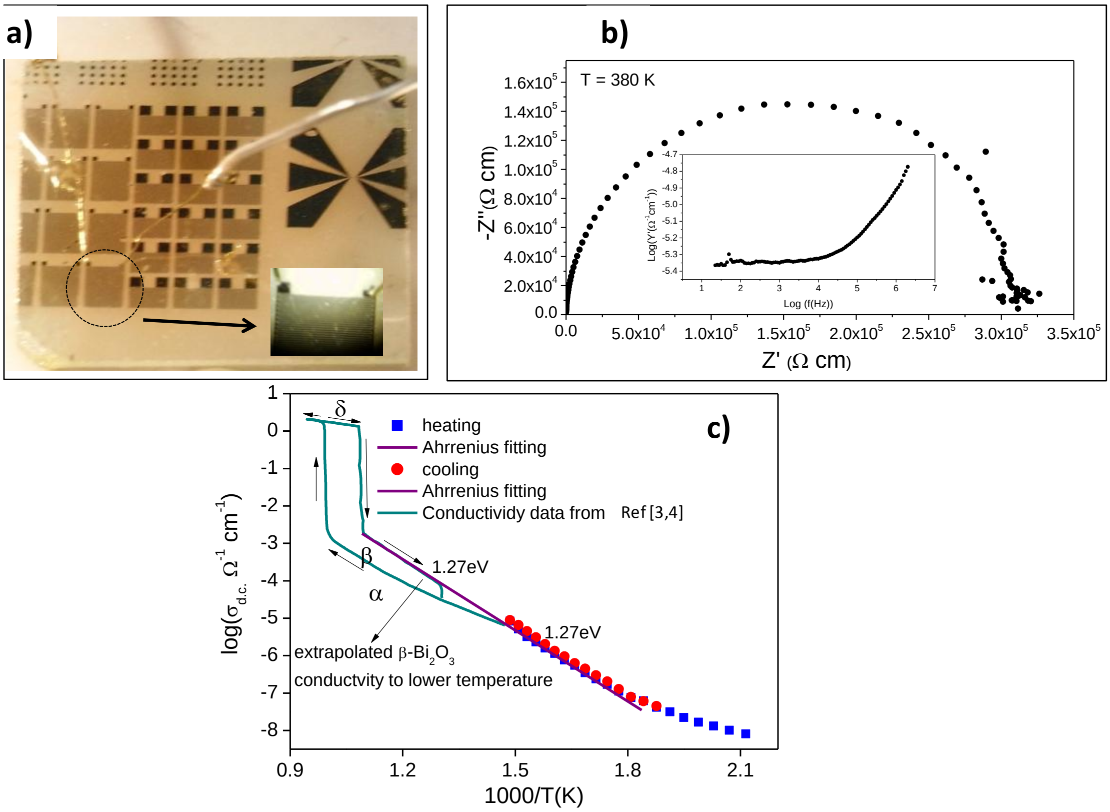

Figure S5. **Conductivity behaviour of β-Bi_2O_3 thin films**. **a)** Experimental set up used to determine the change of the conductivity of the β-Bi_2O_3 film with temperature. The photograph shows a ~0.19 μm thick β-Bi_2O_3 thin film on a Al_2O_3 substrate with interdigital Au electrodes (IDE) on the film surface and the manipulators onto the IDE electrodes pads for making the electric contact. 48 pairs of IDE electrodes of 20 μm width, 1400 μm length and 20 μm separation were deposited on the film surface. **b)** Impedance diagram of the IDE response at 380 K. The lack of electrode blocking at lower frequencies indicates a dominant electronic conductivity. **c)** Conductivity as a function of temperature of the former film.

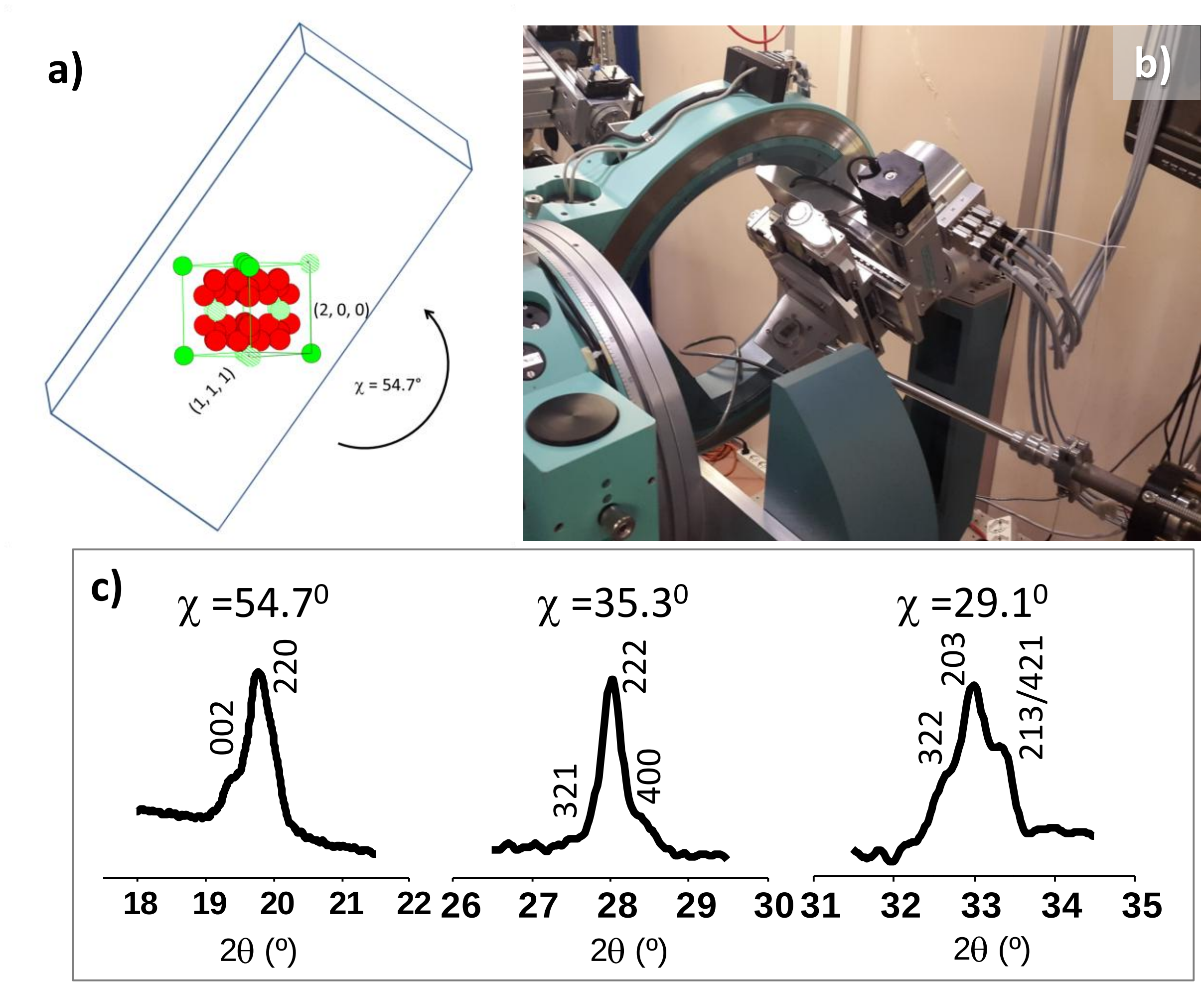

Figure S6. **Details of the XRD measurement at synchrotron facility a)** The film is rotated for the detection of all possible reflections. If we consider that most of the crystals in the film are oriented along <111> perpendicular to the film surface, (200) planes will diffract after the sample is rotated 54.7^0 as the figure indicates. **b)** Photograph of the Eulerian craddle used for the sample rotation. **c)** Detail of the diffraction peaks obtained at different χ and that will be used to obtain a patched XRD pattern of the the Bi_2O_3 film. After determining and subtractting the background level, the integrated area below the diffraction peaks is used in the so-called Currie criterion for peak detection. A peak is considered above detection limits if the intensity is higher than 10 times the background standard deviation. The peaks at the intervals shown are all above this value.

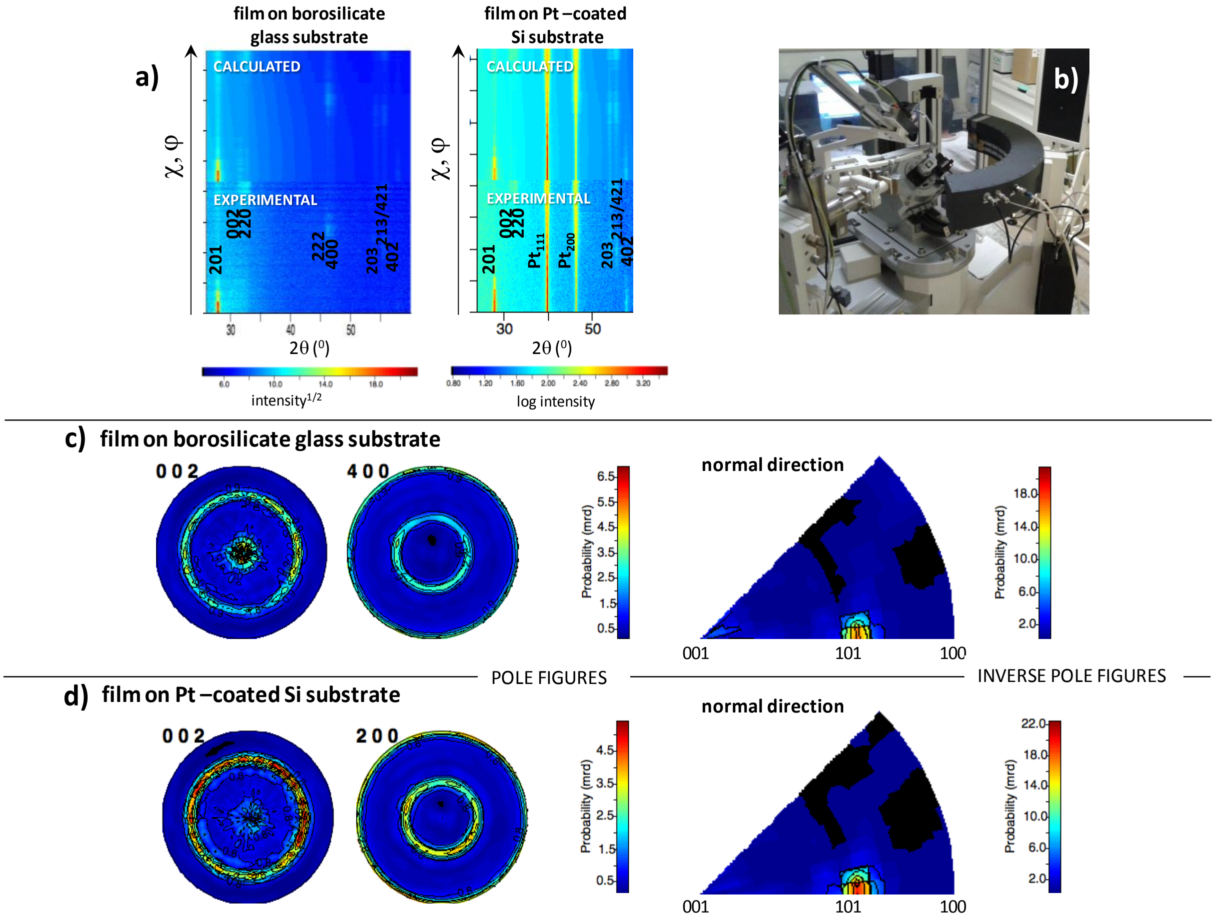

Figure S7. **Details of the analysis of the XRD data obtained for a systematic rotation of the sample** (grid of 5°×5° in χ and φ) **a)** 2D plots of the measured and calculated patterns for different orientations of Bi_2O_3 thin films on borosilicate glass and (111)Pt/TiO_2/SiO_2/(100)Si substrates. Note the absence of significant residual stress (no shift in the position of the peaks with increasing χ). **b)** Diffractrometer set up used **c)** and **d)** Selected pole figures and inverse pole figures calculated from the orientation distribution functions obtained by a combined analysis of XRD data[5] of the films (equal area projection and linear density scale). The results indicate that both films show a similar degree of texture (similar values of multiple of a random distribution, m.r.d., in the pole figures) along a direction close to <201> direction perpendicular to the films surface, according to the corresponding inverse pole figure.

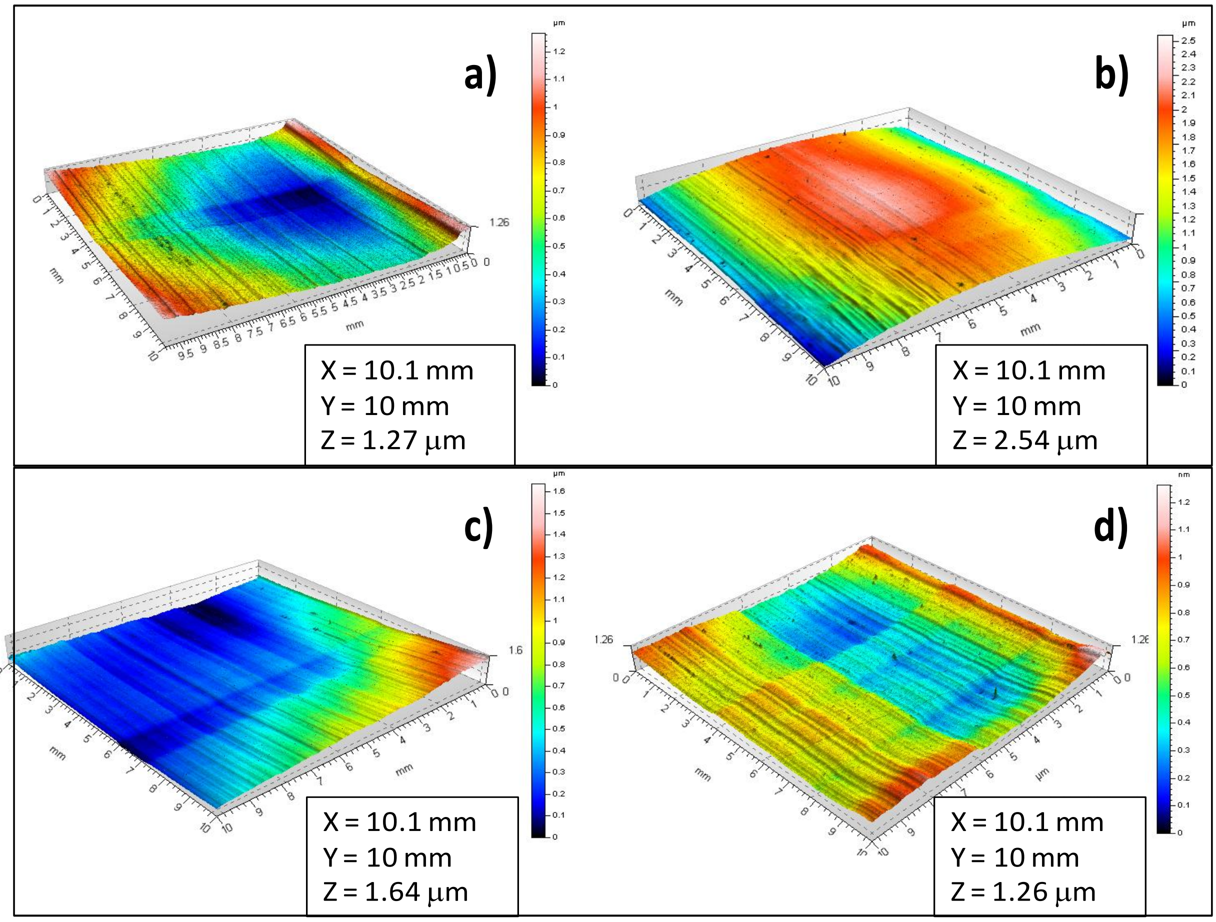

Figure S8. **Images of the surfaces of the substrates and films where the sample curvature was measured by profilemetry**. Stresses of the Bi_2O_3 and β-Bi_2O_3 films were calculated using the modified Stoney equation using the curvature values obtained here. Results are shown in **Table SI . a)** Image of the bare substrate on which the Bi_2O_3 film was subsequently deposited without irradiation, **b)**. **c)** Image of the bare substrate on which the β-Bi_2O_3 thin film was subsequently deposited with irradiation, **d)**.[6, 7]

Table S1. **Stresses of the Bi_2O_3 and β-Bi_2O_3 films deposited on Pt-coated silicon substrates** (films of **Figure 1e(ii) and 1f(ii)**, respectively). R_S and R_f are the curvature radii calculated by profilometry for the bare substrate and the film on the substrate, respectively. t_S and t_f are the substrate and film thickness, respectively. Thickness of the films has been calculated by spectroscopic ellipsometry. Note that the thickness of the β-Bi_2O_3 film is a ~10 % thinner than that of the Bi_2O_3 film (44±1.0 nm and 48±0.5 nm, respectively), which means that bulk density of the former is larger than the latter, as expected from the UV-irradiation.[8] σ corresponds to the calculated stresses using the modified Stoney equation. The absolute error of the calculated stresses taking into account the absolute errors in the measurements of the film thickness and curvature radii is ± 10 %.[6,7] Note that the stresses developed in the β-Bi_2O_3 thin film during processing are almost negligable (the calculated σ value is close to zero taking into account the measurement error). This is contrary to that occurring in the Bi_2O_3 film, where stresses of the order of GPa are developed during the fabrication of the material.

Bi2O3 film				β-Bi2O3 film			
t_f(nm)	R_S(m)	R_f(m)	σ(GPa)*	t_f(nm)	R_S(m)	R_f(m)	σ(MPa)*
48 ± 0.5	32.9 ± 1.7	8.6 ± 0.5	+ 13.0 ± 2.6	44 ±1.0	32.9±1.7	41.7±2.1	– 1.0 ± 0.9

* Stresses are calculated using the modified Stoney equation: $\sigma = [\, E_S / 6(1-\upsilon_S)\,][\, t_S^2/t_f\,][\, 1/R_f - 1/R_S\,]$, where E_S, υ_S and t_S are the Young's module, the Poison ratio and the thicknes of the (100)Si single crystal substrate, respectively. The substrate used here is Pt-coated (100)Si (~1500Å Pt on ~400Å TiO_2 on SiO_2/(100)Si; Radiant Technologies, Inc.). t_f is the thickness of the Bi_2O_3 film. R_S and R_f are the curvature radii of the substrate alone and the substrate with the deposited Bi_2O_3 film, respectively. The Young's module, the Poison ratio and the thickness of the silicon substrate have been considered for these calculations (E_S = 130 GPa, υ_S = 0.3 and $t_S = 0.5 \times 10^3$ nm). Tensile and compressive stresses are identified with "+" and "–" signs, respectively.

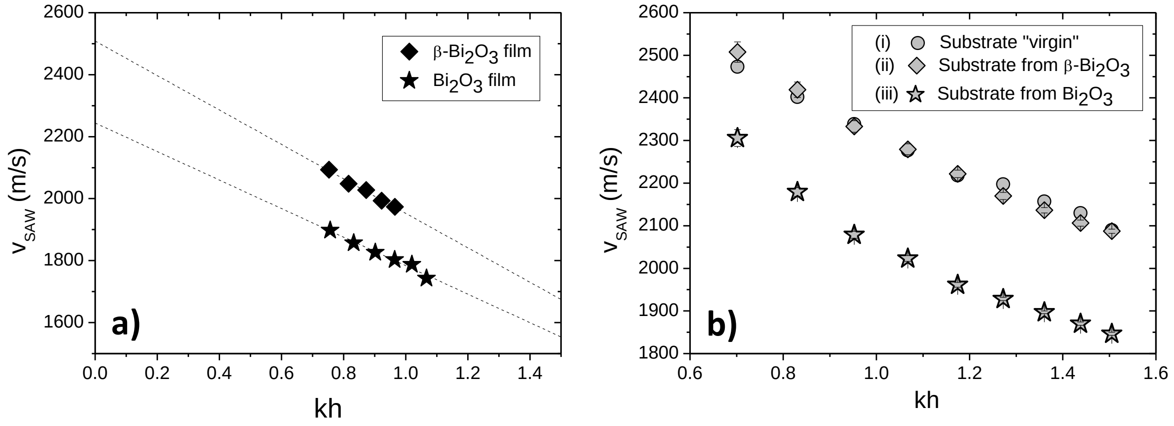

Figure S9. **Brillouin measurements** carried out on **a)** a ~ 48 nm thick Bi_2O_3 film and a ~ 44 nm thick β-Bi_2O_3 film deposited onto Pt-coated silicon substrates (films of **Figure 1e(ii) and 1f(ii)**, respectively). Extrapolated data to zero in the x-coordinate indicate differences in the elastic properties of the substrate due to the different mechanichal stresses generated by the preparation of the pure metastable β-Bi_2O_3 film or the Bi_2O_3 film. These results evidence that both films are different. **b)** Brillouin measurements carried out on bare Pt-coated silicon substrates. Measurements have been made on (i) a "virgin" Pt-coated silicon substrate, which is the original substrate without any bismuth oxide film deposited onto it, (ii) a Pt-coated silicon substrate after removing a ~ 48 nm thick Bi_2O_3 thin film and (iii) a Pt-coated silicon substrate after removing a ~ 44 nm thick β-Bi_2O_3 thin film. Thickness has been calculated from spectroscopic ellipsometry data. These results are indicating that appreciable stresses have not been produced in the preparation of the pure β-Bi_2O_3 film, since the propagation of the sound in its substrate is the same as that of the pristine substrate alone. However, the same measurements carried out in the Bi_2O_3 film's substrate indicate the development of stresses in the sample after the preparation of this film onto the substrate.

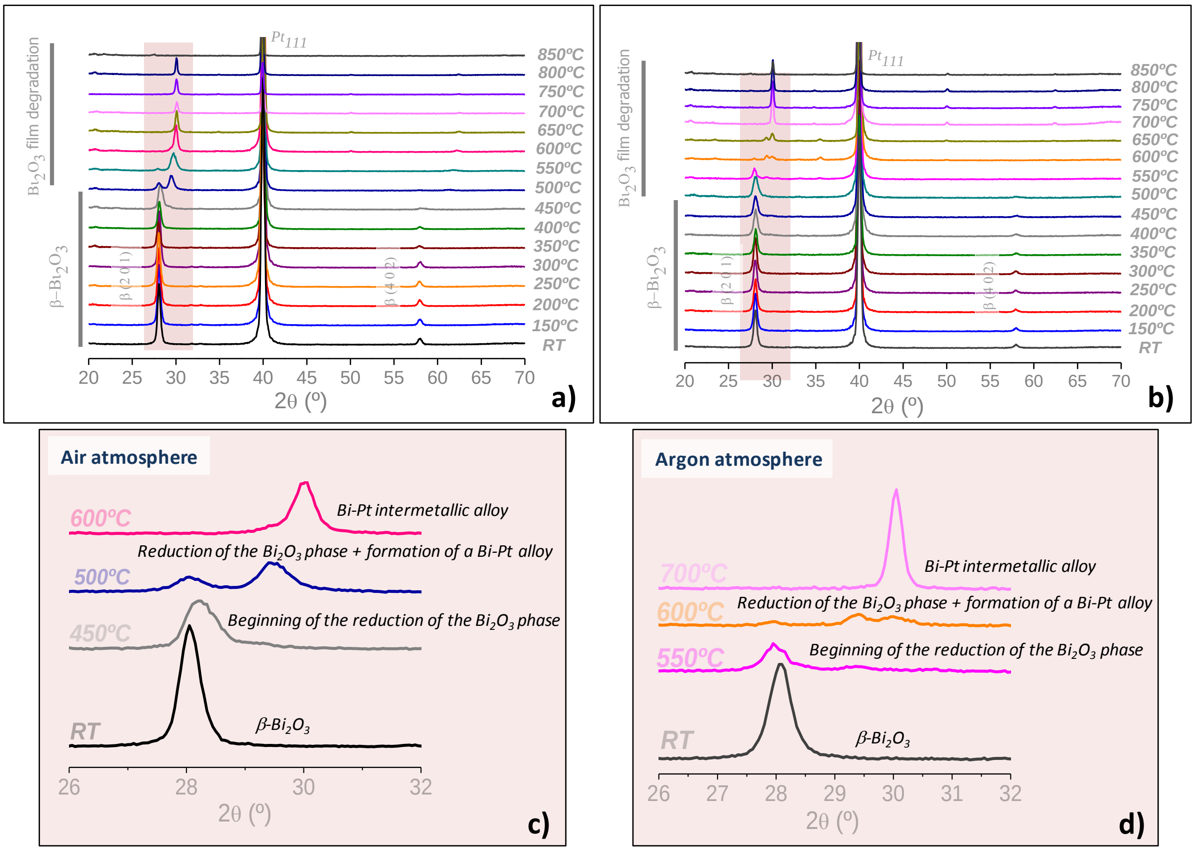

Figure S10. **Stability with temperature of the β-Bi_2O_3 thin films** deposited from UV-absorbing Bi(III) solutions on Pt-coated silicon substrates, irradiated with UV-light (λ=222 nm) for 1 hour at 250°C in oxygen atmosphere and rapid thermal treated in oxygen at 350°C for 1 hour, using a heating rate of 30°C/s. The as-fabricated films show at room temperature the XRD patterns indicated with the label RT in the graphics. Peaks of these RT films can be assigned to the 1, 1, 1 and 2, 2, 2 of the δ-Bi_2O_3 phase or the 2, 0, 1 and 4, 0, 2 of the β-Bi_2O_3 phase. These RT films were subsequently annealed at temperatures between 150°C and 850°C in air [P_{O2}~2×10^{-1} atm] **(a, c)** or in argon [P_{O2}~3×10^{-5} atm, measured for films with ZrO_2 based gauge] **(b,d)**. XRD patterns of the resulting films were obtained in a Siemens D500 powder diffractometer with a Cu anode (λ=1.5406 Å) and a Bragg-Brentano geometry. Measurements were carried out in the 2θ interval between 20º and 70º, with a step of 0.05º per each 3s (a, b). c) and d) correspond to the shaded 2θ intervals in a) and b), respectively, only showing the patterns of those films treated at temperatures where the δ-Bi_2O_3 and/or β-Bi_2O_3 transform into other bismuth containing phases. The JCPDS-ICDD files used for the assignation of the peaks recorded in these patterns are: β-Bi_2O_3 → JCPDS-ICDD-27-0050, δ-Bi_2O_3 → JCPDS-ICDD-27-0052 and Bi_2Pt → JCPDS-ICDD-26-0221. Pt peaks from the (111)Pt/TiO_2/SiO_2/(100)Si substrte are adjusted to the JCPDS-ICDD file 4-0802.

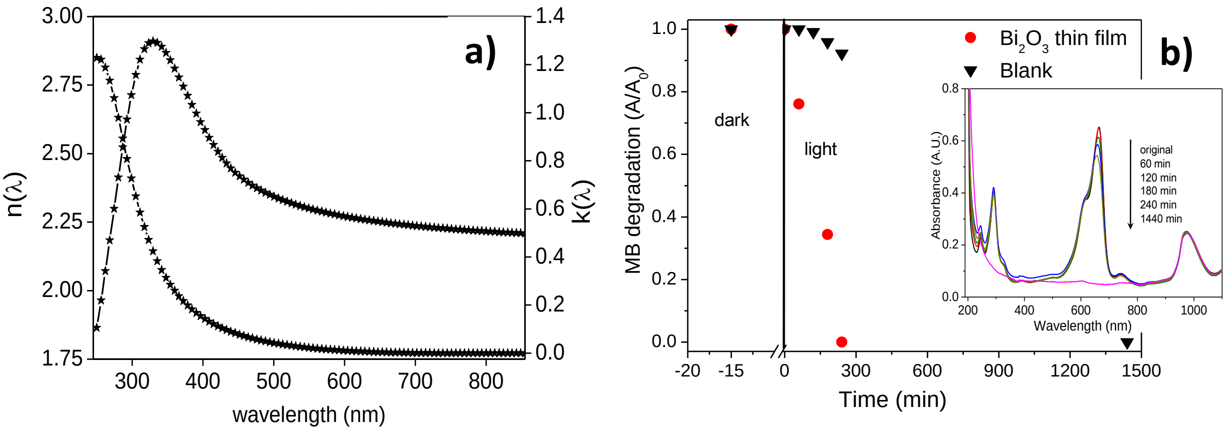

Figure S11. Optical and photocatalytic properties. a) Refractive index, n(λ), and extinction coefficient, k(λ), as a function of the wavelength, λ. b) Photocatalytic degradation kinetics of methylene blue (MB) over a β-Bi_2O_3 thin film on Pt-coated Si substrate, for long times, to show how total degradation of the dye is obtained for times below 300 minutes. The inset shows the photocatalytic degradation of MB by a β-Bi_2O_3 thin film on Pt-coated Si substrate; the peak at ~980 nm corresponds to the absorption of the water used as diluent, so it does not change with the degradation time. The film thickness was ~50 nm and the area of ~2 cm^2

References

1. http://www.icmm.csic.es/eosmad/

2. D.Perez-Mezcua, R.Sirera, R.Jimenez, I.Bretos, C.De Dobbelaere, A.Hardy, M.K. Van Bael and M.L.Calzada. *J.Mater.Chem.C*, **2014**, *2*, 8750.

3. D.L.Proffit. *Ph.D* Thesis, Northwestern University, Evanston, Illinois, USA, December **2013**.

4. H.A.Harwig and A.G.Gerards. *J.Sol.Stat.Chem.*, **1978**, *26*, 265

5. Ricote and D. Chateigner. Quantitative microstructural and texture characterisation by X-ray diffraction of polycrystalline ferroelectric thin films. *J. Appl. Crystall.,* **2004**, 37, 91

6. G.G.Stoney. *Proc.R.Soc.London, Series A*, **1909**, *82*, 172.

7. J.Mendiola, M.L.Calzada, P.Ramos, M.J.Martín and F.Agulló-Rueda. *Thin Solid Films*, **1998**, *315*, 195.

8. H.Imai, H.Hirashima. *J.Ceram.Soc.Jpn.*, **1994**, *102*, 1094.
